# The Role of the Kappa Opioid System in Comorbid Pain and Psychiatric Disorders: Function and Implications

**DOI:** 10.3389/fnins.2021.642493

**Published:** 2021-02-24

**Authors:** Miao-Jin Ji, Jiao Yang, Zhi-Qiang Gao, Liang Zhang, Chao Liu

**Affiliations:** ^1^Jiangsu Province Key Laboratory of Anesthesiology and Jiangsu Province Key Laboratory of Anesthesia and Analgesia Application Technology, School of Anesthesiology, Xuzhou Medical University, Xuzhou, China; ^2^Jiangsu Province Key Laboratory of New Drug Research and Clinical Pharmacy, School of Pharmacy, Xuzhou Medical University, Xuzhou, China; ^3^Department of Neurology, Affiliated Hospital of Qingdao University, Qingdao, China

**Keywords:** kappa opioid system, pain-induced negative affect, cormorbid pain and psychiatric disorders, KOR agonists, KOR antagonists

## Abstract

Both pain and psychiatric disorders, such as anxiety and depression, significantly impact quality of life for the sufferer. The two also share a strong pathological link: chronic pain-induced negative affect drives vulnerability to psychiatric disorders, while patients with comorbid psychiatric disorders tend to experience exacerbated pain. However, the mechanisms responsible for the comorbidity of pain and psychiatric disorders remain unclear. It is well established that the kappa opioid system contributes to depressive and dysphoric states. Emerging studies of chronic pain have revealed the role and mechanisms of the kappa opioid system in pain processing and, in particular, in the associated pathological alteration of affection. Here, we discuss the key findings and summarize compounds acting on the kappa opioid system that are potential candidates for therapeutic strategies against comorbid pain and psychiatric disorders.

## Introduction

Untreated negative affect induced by chronic pain largely drives vulnerability to mood disorders. Epidemiological evidence suggests that the prevalence of depression ranges from 30 to 80% in different pain etiologies (Bair et al., 2003; Howe and Sullivan, 2014), and chronic pain is one of the chief complaints in 65% of patients with treatment-resistant depression (Bair et al., 2003). Moreover, clinical studies have shown that comorbid chronic pain and depression mutually promote disease severity: such patients exhibit a poorer prognosis than those with only one disorder (Scherrer et al., 2016; Sullivan, 2016). Uncovering the mechanisms underlying comorbidity is therefore essential for proper treatment of these patients.

Owing to the complex pathogenesis of comorbid pain and psychiatric disorders, no existing animal model can mimic all of the relevant aspects. However, in recent years, scientists have begun to address the links between the two by dividing pain into sensory and affective dimensions and using behavioral paradigms to analyze affective states in animal models of chronic pain. The results have shown that many types of chronic pain induce aversion phenotypes in animals, including decreased motivation in goal-directed behaviors, conditioned place aversion, longer immobility times in the forced swim test, and decreased time in the light compartment during the light/dark test (Leite-Almeida et al., 2015). These behavioral observations in animals may reflect some of the psychiatric symptoms observed in patients. Thus, uncovering the mechanisms underlying pain-induced aversive states in animals may be the key to understanding the comorbid relationship between chronic pain and mood disorders.

Opioids are the most effective prescription for relieving chronic pain. The classic opioid receptors, the mu opioid receptor (MOR), the delta opioid receptor (DOR), and the kappa opioid receptor (KOR), all belong to the class A (rhodopsin-like) γ-subfamily of seven-transmembrane G protein-coupled receptors (GPCRs), which forms the largest family of targets for current therapeutics. A complete GPCR system involves ligands, a receptor, and transducers. Upon binding to extracellular ligands, GPCRs often undergo a conformational change that causes GDP to be exchanged for GTP bound to Gα, leading to dissociation of Gα and the Gβγ dimer. Both the activated GTP-bound Gα and the Gβγ dimer can transduce signals via second messengers or transducers such as cAMP, inositol trisphosphate (IP_3_), and diacylglycerol (DAG). After agonist activation, GPCRs are phosphorylated by G protein-coupled receptor kinases (GRKs), which recruit arrestin to the original G protein-binding sites. This process makes GPCRs lose the ability to respond to ligand binding, which is referred to as “receptor desensitization.” Long-term use of opioids has been shown to cause addiction and increase the risk of depression (Crofford, 2010; Salas et al., 2017), which may be mediated by the desensitization of opioid receptors. These inherent adverse effects limit the clinical application of opioids. The classic theory of addiction hypothesizes that all addictive drugs enhance dopamine (DA) transmission in the reward circuitry. Interestingly, unlike the excitatory effects of MORs and DORs, KORs normally inhibit neuronal activity and neurotransmission (Tejeda et al., 2013, 2017). Systemic activation of KORs elicits analgesia similar to that induced by MOR activation but with fewer incidences of euphoria and reinforcement. Studies have demonstrated that MORs and KORs have opposite effects on the regulation of motivational processes (Spanagel et al., 1992). These features make KOR a promising drug target to develop non-addictive analgesics.

However, kappa opioid analgesics produce dysphoric effects and psychotomimesis in humans (Walsh et al., 2001; Chartoff and Mavrikaki, 2015) and elicit place aversion and depressive-like affective behaviors in rodents (Chavkin and Koob, 2016; Darcq and Kieffer, 2018). In fact, among the three classical opioid receptor systems, KORs in concert with their primary endogenous ligand, dynorphin, are most heavily implicated in aversion and psychiatric disorders such as depression and anxiety. Increased dynorphin release and KOR expression have been observed in suicidal individuals and preclinical models of neuropsychiatric disorders (Peckys and Hurd, 2001). KOR antagonists are capable of overcoming the pro-depression and anxiogenic effects of chronic or acute stressors (Carr et al., 2010; Browne et al., 2018), and a number of KOR antagonists are already in clinical trials for the treatment of psychiatric disorders (Lowe et al., 2014; Buda et al., 2015; Chavkin and Martinez, 2015). At the same time, KOR expression and function are significantly altered in various chronic pain models, such as peripheral nerve injury (Liu et al., 2019) and chronic constriction injury (Wawrzczak-Bargiela et al., 2020). However, whether the dynorphin/KOR (Dyn/KOR) system is directly involved in pain and psychiatric disorders remains unclear. Considering the opposing effects of KOR activation on chronic pain and depression, how to properly harness KOR agonists as analgesics while avoiding their side effects still needs to be determined. Here, we discuss the function of the Dyn/KOR system and its clinical implications and summarize promising candidates for the treatment of comorbid pain and psychiatric disorders.

## KOR Expression and Signaling

**FIGURE 1:**
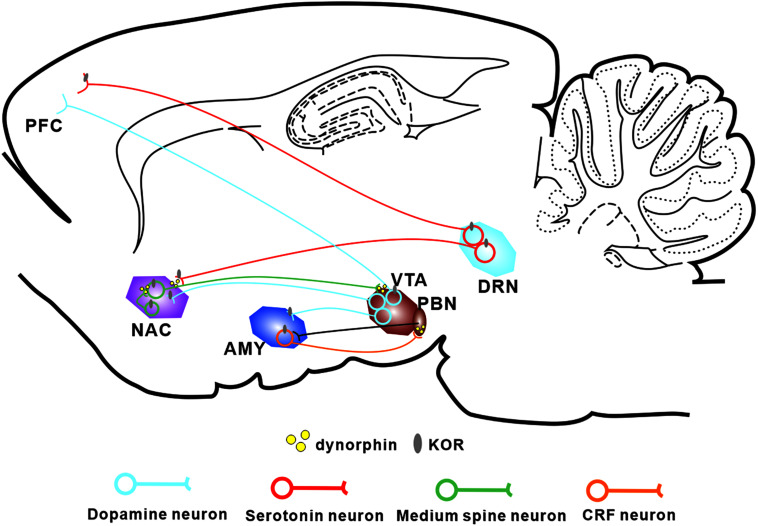
Localization of the Dyn/KOR system within the major circuits involved in pain-induced aversion. KORs are found in major neuronal subtypes in a number of brain areas thought to be involved in pain-induced aversion. KORs modulate the interactions between these circuits through presynaptic and postsynaptic mechanisms. PFC, prefrontal cortex; NAC, nucleus accumbens; AMY, amygdala; VTA, ventral tegmental area; DRN, dorsal raphe nucleus.

The Dyn/KOR system is distributed throughout different brain regions, with KORs expressed in various types of mood-related neurons including serotonergic neurons (Land et al., 2009), corticotropin-releasing factor (CRF) neurons (Marchant et al., 2007), and DA neurons (Liu et al., 2019; Figure 1). It is widely accepted that KOR activation produces negative affect, both in human beings and in rodents (Martinez et al., 2019). Moreover, ablation of KORs from DA neurons or basolateral amygdala (BLA) glutamatergic terminals in the medial prefrontal cortex produces an anxiolytic phenotype (Margolis et al., 2006; Lowery-Gionta et al., 2018), suggesting that the KOR system is critical for the expression of negative affect. Interestingly, besides the regions mentioned above, high levels of the precursor prodynorphin are detected in the periaqueductal gray, the striatum, and the bed nucleus of the stria terminalis (BNST) (Marchant et al., 2007). Given that elements of the Dyn/KOR system are present in the main circuitry involved in both pain processing and affective/motivational systems, it seems likely that the Dyn/KOR system contributes to the aversive nature of chronic pain. At the primary afferent level, KOR is expressed in a transcriptionally distinct subset of peptidergic afferents that strongly express the genes encoding calcitonin gene-related peptide (CGRP) and substance P, and in two populations of low-threshold mechanoreceptors; however, there are very low levels of KORs in cool-sensing neurons and proprioceptors (Snyder et al., 2018).

KORs are located both presynaptically and postsynaptically and play different roles. For example, in the dorsal raphe nucleus (DRN), acute KOR activation inhibits serotonergic neuronal excitability through presynaptic inhibition of excitatory synaptic transmission and postsynaptic activation of ion channels (Lemos et al., 2012). Within the BNST, KORs provide inhibitory control over presynaptic GABAergic signaling (Li et al., 2012; Hwa et al., 2020). Generally, presynaptic KORs modulate monoaminergic and glutamatergic neurotransmitter release whereas postsynaptic KORs hyperpolarize the cell membrane and inhibit neuronal excitation.

**FIGURE 2:**
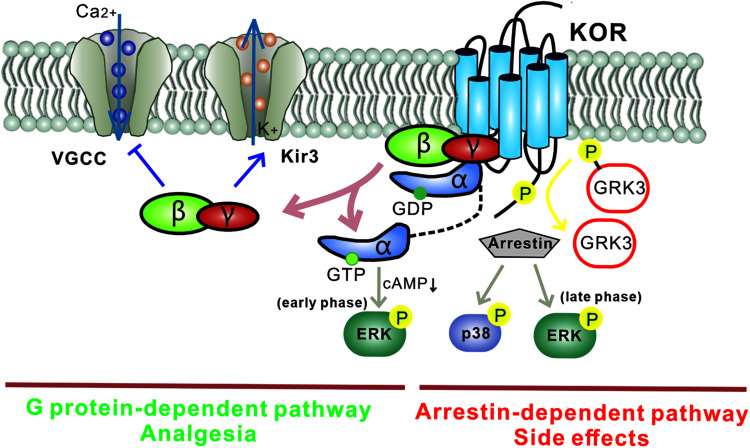
Different downstream KOR signaling pathways mediate distinct functions. The G protein-mediated KOR signaling pathway plays an inhibitory role by activating G protein-gated inwardly rectifying potassium channels (K_*ir*_3s) and blocking voltage-gated Ca^2+^ channels. Gα mediates a decrease in cAMP via Gαi and causes ERK phosphorylation (early phase: 5–15 min after agonist treatment). This classic pathway mediates the analgesia induced by KOR ligands. Separately, KOR activation also recruits arrestin and leads to the activation of several kinase cascades including ERK (late phase: 2 h after agonist treatment) and p38, which mediate the adverse effects induced by KOR ligands. Biased KOR agonism of the G protein-dependent pathway should lead to effective analgesia with fewer side effects. Arrows: activation steps, T lines: inhibition of function. Kir3, G protein-gated inwardly rectifying potassium channel; VGCC, voltage-gated Ca^2+^ channel; P, phosphorylation; cAMP, cyclic adenosine monophosphate; GRK3, G protein-coupled receptor kinase.

Like other GPCRs, most KOR signaling can be divided into two types: classical KOR signal-transduction pathways and KOR-induced arrestin-p38 mitogen-activated protein kinase (arrestin-MAPK) cascades (Figure 2). In the first signaling pathway, Gβγ released from the Gα subunit binds directly to ion channels and plays an inhibitory role by activating G protein-gated inwardly rectifying potassium channels (K_*ir*_3s) or blocking voltage-gated Ca^2+^ channels (Rusin et al., 1997; Sadja et al., 2003). Meanwhile, Gα decreases cAMP and causes ERK phosphorylation. These inhibitory effects have been demonstrated in various cell types ranging from hippocampal granule cells to spinal cord motor neurons, resulting in decreased transmission of nociceptive stimuli at multiple levels of the pain pathways and a profound reduction in the perception of pain. However, several studies have reported excitatory effects of KOR activation, particularly with chronic KOR agonist treatment. This suggests that under some circumstances KORs can activate stimulatory G proteins and upregulate adenylyl cyclase, thus contributing to heterologous tolerance of opioids and physical dependence (Raynor et al., 1994; Avidor-Reiss et al., 1997).

In the second signaling pathway, KORs recruit β-arrestin scaffolding proteins after agonist-induced GRK3 phosphorylation of the C-terminal intracellular domain. It has been reported that phosphorylation at KOR serine 369 in rodents (Land et al., 2009) or serine 358 in humans (Li et al., 2002) leads to KOR desensitization and internalization. The recruited arrestin mediates p38 MAPK and ERK1/2 phosphorylation (Bruchas et al., 2008; McLennan et al., 2008) and has been observed to occur following behavioral stress. The β-arrestin signaling pathway has been demonstrated to contribute to KOR-induced aversion, dysphoria, and sedation, but not to KOR-induced analgesia. Thus, functionally selective KOR agonists, termed “biased agonists,” may be able to selectively avoid p38 MAPK activation and hold promise for pain relief without side effects (as detailed in the section “Drug Candidates and Future Directions,” below). Possible substrates of the KOR-activated p38 MAPK pathway include ion channels (Nav1.8, Kir3.1), serotonin transporters (SERT), and transcription factors (zif268, elF4B) (Hudmon et al., 2008; Steiner et al., 2008; Guan et al., 2010; Ruan et al., 2010). Regulation of these effectors may explain the characteristics of the behavioral responses seen with KOR-induced p38 activation. Arrestin-dependent KOR-induced ERK1/2 phosphorylation occurs 2 h after agonist treatment and is known as late-phase ERK1/2 phosphorylation; early phase ERK1/2 activation occurs 5–15 min after agonist treatment and depends on the Gβγ subunit. Potential effects of KOR-induced early phase ERK1/2 activation include increases in AMPA receptor cell surface expression, growth of dendritic spines, and regulation of CREB activation. Interestingly, KORs can activate different signaling cascades within a single brain region (Hjelmstad and Fields, 2003).

## Dyn/KOR and the Neural Circuits Involved in Pain-Induced Negative Affect

### Mesolimbic Circuitry

Recent studies on pain and depression show that decreased motivation in goal-directed behavior is a characteristic feature of pain-induced negative affect (Cahill et al., 2014; Ji et al., 2018; Liu et al., 2019). Thus, the mesolimbic circuitry, which is also known as the reward circuitry, may play a critical role in driving pain-induced negative affective states. This circuitry is composed of DA neurons in the ventral tegmental area (VTA) and their projections to forebrain limbic structures such as the nucleus accumbens (NAc) and prefrontal cortex (PFC).

VTA DA neurons receive strong dynorphin projections from the striatum, the lateral hypothalamus, the central nucleus of the amygdala (CeA), and the BNST (Fallon et al., 1985; Kaufling et al., 2017). Recently, Cahill’s lab reported that chronic pain caused an increase in KOR mRNA (Oprk1) expression in DA neurons only in male mice and that pain-induced aversive states were increased by a KOR agonist in male but not female mice (Liu et al., 2019). The authors attributed the observed sex differences to complex hormonal and sex-chromosome factors, which are known to influence the depressive and anti-nociceptive effects of KOR in non-pain models (Russell et al., 2014; Abraham et al., 2018; Zhang et al., 2018). Liu et al. (2019) also reported that ablation of KORs from DA neurons using AAV-TH-cre virus in KOR loxP mice prevented pain-induced aversive states without affecting the sensory dimension of chronic pain (Liu et al., 2019). KORs are distributed in both the somatodendritic and terminal regions of VTA DA neurons (Tejeda et al., 2013). Activation of KORs located in the terminals of DA neurons inhibits DA release in efferents (Spanagel et al., 1992), but activation of KORs located in VTA cell bodies appears to have pathway-dependent effects, hyperpolarizing the dopaminergic projection to the mPFC and amygdala but not the projection to the NAc core (Margolis et al., 2006, 2008). Circuit DA dynamics are also shaped by Dyn/KOR-induced presynaptic inhibition of excitatory neurotransmission and GABA release onto VTA DA neurons (Graziane et al., 2013). Although many findings indicate that inhibition of the DA output is the mechanism underlying KOR agonist-induced aversion, there is evidence to suggest that the recruitment of p38 MAPK signaling in DA neurons is critical. Conditional knockout of p38 signaling in DA neurons did not abolish the ability of a KOR agonist to inhibit DA release but did block KOR-mediated aversion (Ehrich et al., 2015). Indeed, DA release is not decreased in many pain paradigms (Navratilova et al., 2012; Xie et al., 2014), such as PGE2-induced hyperalgesia (Vergara et al., 2020), that produce aversive affect in behavioral tests. Therefore, the hypothesis that the recruitment of p38 MAPK signaling in DA neurons is more important than DA transmission alteration in KOR-mediated aversion seems to work in pain states, but further work is needed to identify the mechanism underlying the effects of the VTA Dyn/KOR system in pain-induced negative emotions.

Unlike in the VTA, which receives dynorphin projections from other nuclei, dynorphin is released locally in the NAc from medium spine neurons (MSNs). Massaly et al. (2019) reported an increase in local dynorphin tone in the NAc shell in inflammatory pain, resulting from pain-induced selective disinhibition of dynorphin-containing neurons. Similarly, higher levels of Oprk1 mRNA are observed in the NAc in animals with a spared nerve injury (Palmisano et al., 2017). It is noteworthy that the increase in KORs usually occurs during the early phase of neuropathy whereas downregulation of Oprk1 gene expression happens during the late phase, 2 weeks after surgery (Vergara et al., 2020), and may be attributed to internalization and densensitization of KORs. Furthermore, it has been demonstrated recently that KOR blockade in the NAc reverses preclinical measures of injury-induced aversion and anhedonia and that photogenetic activation of dynorphin-containing MSNs is sufficient to lead to negative affective states (Massaly et al., 2019). In paclitaxel-induced neuropathy, an injury-free model, a KOR antagonist injected into NAc reversed paclitaxel-induced anhedonia but not mechanical hypersensitivity (Meade et al., 2020). Together, these results suggest that pain-induced negative affect is mediated via recruitment of the NAc Dyn/KOR system.

### Amygdala

The amygdala circuitry most relevant for pain-related functions includes the BLA, the central nucleus of the amygdala (CeA), and the intercalated cell clusters interposed between them (Thompson and Neugebauer, 2017, 2019). KORs are located on cells in both the BLA and the CeA, and dynorphin is released from distal projections as well as synthesized locally in the lateral subdivision of the CeA (CeL) (Kravets et al., 2015).

Previous studies have demonstrated that chronic pain promotes neuroplasticity mainly in the CeA (Carrasquillo and Gereau, 2008). Interestingly, modulation of the Dyn/KOR system in the CeA induces significant changes in pain-induced aversion (Nation et al., 2018; Navratilova et al., 2019) and KORs in the CeA have been linked to negative affective states associated with ongoing pain. Microinjection of a long-lasting KOR antagonist, nor-binaltorphimine (nor-BNI) into the right CeA before spinal nerve ligation in rats prevented neuropathic-induced conditioned place preference to intravenous gabapentin, suggesting that nor-BNI eliminated the aversiveness of ongoing pain (Nation et al., 2018). This effect was mediated by blocking the KOR-mediated disinhibition of CeA output neurons involved in neuropathic pain. Similarly, activation of the pathway from the lateral parabrachial nucleus (lPBN) to the CeA generates an aversive memory and dynorphin-expressing neurons are required in the process (Chiang et al., 2020). The lPBN is a major target of spinal projection neurons conveying nociceptive input to supraspinal structures. Most of the outputs from the CeA are GABAergic, coexpressing CRF and dynorphin, and project back to the lPBN. Optogenetic stimulation of the CeA–lPBN pathway suppresses acute pain and inhibiting it evokes pain behaviors in naïve animals, but the efficacy of this pathway is suppressed in chronic pain states (Raver et al., 2020). Hence, the CeA–lPBN circuit plays a negative feedback role in response to noxious stimuli under normal conditions but becomes inefficient in chronic pain states, and the Dyn/KOR system in the CeA is necessary for this role.

### Dorsal Raphe Nucleus

The serotonergic system has been the focus of many studies on the relationship between pain and depression. The dorsal raphe nucleus (DRN) is one of the major sources of serotonin (5-HT) in the brain. Administration of complete Freund’s adjuvant (CFA) results in sustained inflammatory pain and leads to depression-like behaviors. This model is widely used to induce comorbid pain and depression, which is characterized by depletion of 5-HT and its metabolism-related precursors in the brain (Zhang et al., 2016). Similarly, decreased levels of 5-HT are observed in the PFC in rats infused with a cocktail of inflammatory agents into the dura mater, which induces chronic headache and anxiodepressive-like behaviors (Zhang et al., 2017). Furthermore, recent human functional magnetic resonance imaging data show that functional connectivity between the DRN and the CeA is reduced in patients with comorbid depressive symptoms but not in patients with chronic pain only, compared with healthy controls (Zhou et al., 2019). Pharmacological and optogenetic results in animals further implicate a novel pathway involving 5-HT projections from the DRN to somatostatin-expressing neurons in the CeA in the comorbidity (Zhou et al., 2019).

Previous work indicates that administration of a KOR agonist into the DRN decreases extracellular 5-HT by approximately 30% (Fuentealba et al., 2010). It has been demonstrated that the Dyn/KOR system modulates serotonin transmission, especially in stress-related behaviors. First, KOR activation in the DRN inhibits the excitatory inputs onto serotoninergic neurons. Second, KORs increase postsynaptic G protein-gated inwardly rectifying potassium channel (GIRK) currents in the DRN. Lastly, KORs mediate the translocation of the serotonin transporter SERT via a p38 MAPK-dependent mechanism. Moreover, repeated stress exposure induces dynorphin release and KOR activation in 5-HT neurons in the DRN (Lemos et al., 2012). As a form of repeated stress, chronic pain may alter the DRN–CeA pathway by regulating the Dyn/KOR system in the DRN; however, more clinical and *in vivo* evidence is needed to fully elucidate the underlying mechanisms.

## Drug Candidates and Future Directions

There is no doubt that an analgesic with antidepressant and/or anxiolytic effects is optimal for patients with comorbid pain and mood disorders. However, this has proved to be an elusive goal for clinical and laboratory researchers for many decades. As one of the most commonly used categories of analgesic, opioids and opioid-based therapies may be the key to achieving this goal. Indeed, progress has already been made on several specific compounds targeting KORs for treatment of comorbid pain and psychiatric disorders (detailed in Table 1).

**TABLE 1:** Potential compounds for the treatment of comorbid pain and psychiatric disorders.

Compound	Function	Subjects and dose: pain treatment	Pain model/paradigm	Subjects and dose: aversion test	Aversive behavioral paradigm	References
Nalfurafine (TRK-820)	G-protein-biased KOR agonist	C57BL/6 mice 15 μg/kg	Warm-water tail-withdrawal			
Mesyl SalB	G-protein-biased KOR agonist	B6.SJL mice 1 mg/kg	Intraplantar formaldehyde (inflammatory pain model), warm-water tail-withdrawal	SD rat 0.3 mg/kg	Sucrose self-administration CPA CTA	Kivell et al., 2018
EOM SalB	G-protein-biased KOR agonist	C57BL/6J mice 0.1, 0.3 mg/kg	Warm-water tail-withdrawal	SD rat 0.1, 0.3 mg/kg	CPA EPM FST	Ewald et al., 2017; Paton et al., 2017
RB-64	G-protein-biased KOR agonist	C57BL/6 mice 3, 10 mg/kg	Hotplate analgesia	C57BL/6 mice 3 mg/kg	CPA	White et al., 2014; White et al., 2015
HS665	G-protein biased KOR agonist	C57BL/6J mice 10, 30 nmol, i. c.v.	Warm-water tail-withdrawal	C57BL/6J mice 10, 30 nmol, i.c.v.	CPA (have aversion)	Spetea et al., 2017
		C57BL/6J mice 30 nmol, i. c.v.	Acetic acid-induced writhing			Spetea et al., 2012
HS666	G-protein-biased KOR agonist	C57BL/6J mice 10, 30 nmol, i. c.v.	Acetic acid-induced writhing	C57BL/6J mice 30 nmol, i.c.v.	CPA	Spetea et al., 2012
Collybolide	G-protein-biased KOR agonist	C57BL/6J mice 2 mg/kg	Tail flick test	C57BL/6J mice 2 mg/kg	CPA FST EPM	Gupta et al., 2016
Difelikefalin (CR845)	Peripherally restricted KOR agonist	C57BL/6J mice 10 mg/kg	Spinal nerve ligation (neuropathic pain model)	C57BL/6J mice 10 mg/kg	OFT	Beck et al., 2019a
		Human clinical III phase	Postoperative pain			Gardell et al., 2008
JNJ-38488502 (CR665)	Peripherally restricted KOR agonist	SD rat 20 mg/kg	Acetic acid-induced writhing. hot plate analgesia	SD rat 20 mg/kg	OFT	Hughes et al., 2013
JT09	Peripherally restricted KOR agonist	SD rat 10 mg/kg	Acetic acid-induced writhing. hot plate analgesia	SD rat 20 mg/kg	CPA FST	Beck et al., 2019b
Nor-BNI	KOR antagonist	C57BL/6J mice 3 mg/kg, s.c 2.5 μg/lateral	Cephalic and extracephalic cutaneous allodynia, tail flick test	C57BL/6J mice SD rat	CPA FST EPM	Xie et al., 2017; Page et al., 2019
CYM-51317	KOR antagonist	C57BL/6J mice 20 mg/kg 1 μg/lateral	Cephalic and extracephalic cutaneous allodynia, tail flick test			Xie et al., 2017
CYM-53093	KOR antagonist	C57BL/6J mice 10 mg/kg	Tail flick-test migraine			Guerrero et al., 2019
Buprenorphine	Partial MOR agonist and KOR antagonist	Human (1–4 mg/d)	Postoperative moderate to severe pain	Older adults 0.4 mg/d	Montgomery–Asberg Depression Rating scale	Karp et al., 2014; Yovell et al., 2016

*CPA, conditioned place aversion; CTA, conditioned taste aversion; EPM, elevated plus maze; FST, forced swim test.*

### Biased G Protein KOR Agonists

Upon extracellular ligand binding, GPCRs usually undergo a conformational change that activates heterotrimeric G proteins, a process that is important for transmitting the required signals. The ability of agonists acting at the same GPCR to preferentially elicit different signaling pathways by stabilizing the receptor in a particular active conformational state is called “biased agonism” or “functional selectivity.” The discovery of this phenomenon offers a therapeutic alternative to conventional full KOR agonism, which provides effective analgesia but at the cost of significant side effects including dysphoria, sedation, anxiety, and depression. There is consensus that KOR-coupled G protein signaling is the major pathway for the analgesic effects of KOR agonists whereas the arrestin-p38 MAPK cascade is required for aversion and other effects (Fields, 2011). Thus, biased G protein KOR agonists are promising compounds for the treatment of chronic pain.

Nalfurafine (TRK-820) was the first biased KOR agonist used in a clinical setting, for medication-resistant pruritus in hemodialysis patients (Kumagai et al., 2010). Recently, it has been reported that co-administration of nalfurafine with morphine beneficially modulates both the analgesic and rewarding properties of morphine in mice. Moreover, the dose of nalfurafine that produced a significant effect in the preclinical study (15 μg/kg) was similar to the antipruritic dose in mice, suggesting that the clinical dose may provide adequate analgesic synergy while avoiding significant antitherapeutic effects (Schattauer et al., 2017). Modification of the structural scaffold of salvinorin A (SalA), a potent KOR agonist, has produced several biased ligands, such as mesyl SalB, ethoxymethyl ether SalB (EOM SalB), and 22-thiocyanatosalvinorin A (RB-64). These are better biased G protein agonists for human KOR than SalA or U50,488H, another balanced KOR agonist (Simonson et al., 2015; Kivell et al., 2018). Mesyl SalB and EOM SalB produce longer-lasting analgesia in a warm-water tail-withdrawal rodent assay than SalA, without aversion, anxiety, or depressive-like effects (Ewald et al., 2017; Paton et al., 2017). Similarly, RB-64 induced analgesia in a hotplate assay in both wild-type and β-arrestin2 knock-out mice and, at a low dose, did not cause aversion-like responses (White et al., 2015). HS665 and HS666, which are diphenethylamine derivatives, exhibit great affinity for KORs and very weak partial agonism for β-arrestin-2 signaling. These compounds elicited a potent dose-dependent analgesic effect in the warm-water tail-withdrawal assay when administrated intracerebroventricularly (Spetea et al., 2012, 2017) and had no effect on locomotor behavior or aversion (Erli et al., 2017). A different study showed that both agents display analgesic action in an acetic acid writhing assay when injected subcutaneously, while demonstrating no motor impairments or sedation (Dunn et al., 2019). Collybolide (Colly) is a very potent biased agonist for treating comorbid pain and psychiatric disorders. Similar to SalA, it has an antinociceptive effect in the tail-flick assay and produces some aversion. However, unlike SalA, Colly exhibits slight antidepressant and anxiogenic effects in the forced swim and open-field tests (Gupta et al., 2016).

### Peripherally Restricted KOR Agonists

Activation of peripheral KORs alone can produce a significant analgesic effect. Thus, the development of peripherally restricted KOR agonists that cannot cross the blood–brain barrier is a viable strategy for avoiding the side effects associated with activation of KORs in the CNS. However, many of the early compounds were ruled out because of insufficient antinociception; researchers were unable to identify effective compounds until recently.

Difelikefalin (CR845) and JNJ-38488502 (CR665) are peripherally restricted tetrapeptide KOR agonists that have shown promising results in preclinical studies, including a reduction in writhing behaviors and inflammatory pain in animal models (Hughes et al., 2013). CR845 is also effective in relieving abdominal pain and mechanical allodynia in a spinal nerve ligation model of neuropathic pain (Beck et al., 2019a). CR845 is currently in phase III clinical trials for the treatment of postoperative pain and uremic pruritus. One of the derivatives of CR665, termed JT09, is currently in development by JT Pharmaceuticals. 20 mg/kg JT09 administered via oral gavage in rats exhibited similar analgesic effects as 10 mg/kg morphine, but JT09 showed no sedative or pro-depressive effects in behavioral tests (Beck et al., 2019b). The pharmacodynamics of JT09 and its antinociceptive effects in chronic pain models are still being investigated. There is also a range of peripherally restricted derivatives of nalfurafine that have an increased number of hydrogen bond donors, and these have yielded promising results. These compounds produce dose-dependent anti-allodynic effects in the acetic acid writhing mouse model (Suzuki et al., 2017). Further work is planned to fully evaluate the antinociceptive potential of these compounds and assess the side effects. However, whether peripherally restricted KOR agonists can block pain-related depression is still unclear.

### KOR Antagonists

Although it is well known that blockade of KOR activation prevents stress-induced aversive affects, the potential clinical use of KOR antagonists as antidepressants was not addressed until recently. Interestingly, KOR antagonists have been found to play an analgesic role in several injury-free pain studies. For example, stress produces allodynia in many injury-free models of cephalic pain. Microinjections of nor-BNI or CYM-51317 (a novel short-acting KOR antagonist) into the CeA in rats prevented this stress-induced allodynia (Xie et al., 2017). Similarly, oral administration of CYM-53093 had a protective effect against migraine (Guerrero et al., 2019).

Using compounds which are both antagonistic to KORs and agonistic to other opioid receptors may be another good choice for treating comorbid pain and psychiatric disorders. Buprenorphine, a partial MOR agonist and KOR antagonist that provides long-lasting analgesia for chronic pain, is a promising candidate. It has been investigated for its anti-depression potential in preclinical studies and clinical trials and produced a rapid and sustained improvement in elders with resistant depression, even at a low dose. Furthermore, an early open-label study reported that buprenorphine alleviates negative symptoms in patients with treatment-refractory, unipolar, non-psychotic, major depression (Karp et al., 2014; Yovell et al., 2016).

## Conclusion

Over the past decades, information has accumulated about the pathophysiological and pharmacological implications of the role of the Dyn/KOR system in the comorbidity of chronic pain and mood disorders. The Dyn/KOR system mediates pain-induced aversive states by regulating many aspects of emotion processing, including DA neurotransmission of the mesolimbic circuitry, the efficacy of the CeA–lPBN pathway, and the intrinsic excitability of 5-HT neurons. A new generation of biased KOR agonists together with new clinical medication strategies has led to a focus on KORs as a potential drug target for pain and psychiatric disorders with fewer side effects. These studies have increased our understanding of how the Dyn/KOR system is involved in pain and mood disorders and have revealed promising therapeutic targets for the treatment of comorbid pain and psychiatric disorders.

## Author Contributions

M-JJ, JY, and Z-QG drafted the manuscript. CL critically edited the manuscript. LZ contributed substantially to the manuscript revision. All authors approved the manuscript in its final form.

## Conflict of Interest

The authors declare that the research was conducted in the absence of any commercial or financial relationships that could be construed as a potential conflict of interest.
